# Population, workforce, and organisational characteristics affecting appointment rates: a retrospective cross-sectional analysis in primary care

**DOI:** 10.3399/BJGP.2022.0625

**Published:** 2023-08-22

**Authors:** Tianchang Zhao, Rachel Meacock, Matt Sutton

**Affiliations:** Policy and Economics, School of Health Sciences, University of Manchester, Manchester.; Policy and Economics, School of Health Sciences, University of Manchester, Manchester.; Policy and Economics, School of Health Sciences, University of Manchester, Manchester.

**Keywords:** access, activity, consultations, deprivation, population, primary health care, workforce

## Abstract

**Background:**

The recent publication of data on appointment volumes for all general practices in England has enabled representative analysis of factors affecting appointment activity rates for the first time.

**Aim:**

To identify population, workforce, and organisational predictors of practice variations in appointment volume.

**Design and setting:**

A multivariable cross-sectional regression analysis of 6284 general practices in England was undertaken using data from August–October 2022.

**Method:**

Multivariable regression analyses was conducted. It related population age and deprivation, numbers of GPs, nurses, and other care professionals, and organisation characteristics to numbers of appointments by staff type and to proportions of appointments on the same or next day after booking.

**Results:**

Appointment levels were higher at practices serving rural areas. Practices serving more deprived populations had more appointments with other care professionals but not GPs. One additional full-time equivalent (FTE) GP was associated with an extra 175 appointments over 3 months. Additional FTEs of other staff types were associated with larger differences in appointment rates (367 appointments per additional nurse and 218 appointments per additional other care professional over 3 months). There was evidence of substitution between staff types in appointment provision. Levels of staffing were not associated with proportions of same-or next-day appointments.

**Conclusion:**

Higher staffing levels are associated with more appointment provision, but not speed of appointment availability. New information on activity levels has shown evidence of substitution between GPs and other care professionals in appointment provision and demonstrated additional workload for practices serving deprived and rural areas.

## INTRODUCTION

Primary care is in crisis, with workload pressures becoming unsustainable for many practices.^1^^,^^2^ GPs have the highest rates of burnout and turnover of all medical specialties.^3^ In England, patient-reported satisfaction with access is at an all-time low despite record numbers of appointments being provided.^4^ Increasing access is therefore a key policy priority. The government has pledged to increase appointment numbers by widening the types of staff that can work in general practice.^5^ However, there is little evidence to support this strategy.^6^ The degree to which other healthcare practitioners can act as substitutes for GPs, and the contribution of different staff types to overall appointment volumes, is unknown.

Recent research has examined associations between workforce composition and several outcomes of importance to patients, staff, and the health system. The number of GPs was found to relate positively to most outcomes.^6^^,^^7^ However, numbers of other care professionals were negatively associated with patient-reported access and satisfaction. Other types of health professionals were not found to be substitutes for GPs in terms of outcomes and employing clinicians who were not GPs did not reduce GPs’ workloads.^8^ However, only proxy measures of patient-reported access were examined, such as the time since patients reported last having an appointment. The associations between workforce composition and organisational factors with activity in terms of appointment volumes is unknown. This is critical information for addressing the current primary care crisis.

This study examined newly published data on volumes of appointments provided by general practices in England. The aim was to identify population, workforce, and organisational predictors of practice variations in the volume of appointments provided, which is a direct measure of patient access.

## METHOD

### Data

Practice-level data on appointments were obtained from August–October 2022. These were recently published for the first time.^9^ The data series contains monthly counts of attended appointments at each practice by staff type (classified as GP or other care professional), and time between booking and appointment, covering 98.7% of practices and 99.8% of all registered patients in England. The grouping of other care professionals covers a wide range of staff including dispensers, link workers, and practice nurses.^10^ To minimise potential effects of seasonality and volatility, especially as the first release of these experimental data are used, the 3 months of available data were aggregated.

Information was also obtained on practices’ registered populations and their age composition, and full-time equivalent (FTE) numbers of GPs, nurses, other direct patient care professionals, and administrative staff employed as of October 2022 from NHS Digital’s General Practice Workforce data.^11^ Both the appointments and workforce data are publicly available from the NHS Digital website. The appointments data can be examined visually in the NHS Digital interactive tool at aggregated levels.^9^

**Table 1:** How this fits in

Supply and demand predictors of variation in activity levels for all practices in England could not be identified until now. This study has shown that appointment rates per person are higher for practices serving rural areas. Appointment rates with other care professionals are higher in deprived areas but appointment rates with GPs are not. There is clear evidence of substitution between GPs and other care professionals in the provision of appointments.

Information on the geographical distribution of registered populations was used from the Patients Registered at a GP Practice dataset,^12^ linked to the most recent indices of deprivation for Lower-layer Super Output Areas (LSOAs),^13^ to calculate population-weighted mean overall Index of Multiple Deprivation (IMD) scores for each practice, based on their registered populations’ area of residence. Practices were then assigned to deprivation quintiles, based on these mean overall IMD scores.

In addition, the following practice characteristics sourced from the NHS Payments to General Practice dataset were included: rurality; contract type; and dispensing status.^14^

Total appointment volumes, and volumes of appointments with GPs and other care professionals, per 1000 registered patients, were calculated. Using the data on time between booking and appointment, proportions of appointments that took place on the same or next day of booking were also calculated.

Appointment volumes were available for all 3 months for 6391 practices in England. The following exclusions were made: 18 practices with <1000 registered patients, as these were likely to be serving specific populations or were in the process of opening or closing; nine practices where workforce data were not available; 74 practices with ‘atypical characteristics’, such as large fluctuations in patient numbers or part-year data only (as classified in the NHS Payments to General Practice dataset); and six practices for which payments data were not available. A total of 6284 practices were included in the final analysis.

### Analysis

First, practice variation in the number of appointments provided per 1000 patients was analysed. Linear regression was used to estimate associations with FTE numbers of each staff group per 1000 registered population, population characteristics (age composition and deprivation), and practice characteristics (rural location, contract type, and dispensing status). These analyses were undertaken first for all appointments, and then for appointments with GPs and with other care professionals separately. As staff type is reported as unknown for some appointments, the separate figures do not sum to the total.

Variation in the proportion of appointments that are seen on the same or next day of booking were then examined. Total appointments (with any type of staff) were considered first, and then appointments with GPs and appointments with other care professionals were considered separately. This is because appointments with different staff members may differ in urgency and the degree to which they are scheduled further in advance.

In the main analysis, practices with total appointment rates below the 1^st^ percentile or above the 99^th^ percentile were excluded to minimise the influence of outliers. The sensitivity of the results to this was checked in supplementary analysis. For the analysis of appointment rates with specific staff, a supplementary analysis was undertaken in which practices with >10% missing information on staff type were excluded. For the analysis of proportion of appointments seen shortly after booking, a supplementary analysis was performed whereby practices for which this information was unknown for >1% of appointments were excluded.

All analysis was undertaken in Stata (version 17). Regressions were weighted by the denominator of the dependent variable (registered population for appointment rates and volume of appointments for proportion of appointments seen shortly after booking). Standard errors were adjusted for heteroscedasticity using Stata robust option.

## RESULTS

**Figure 1.**
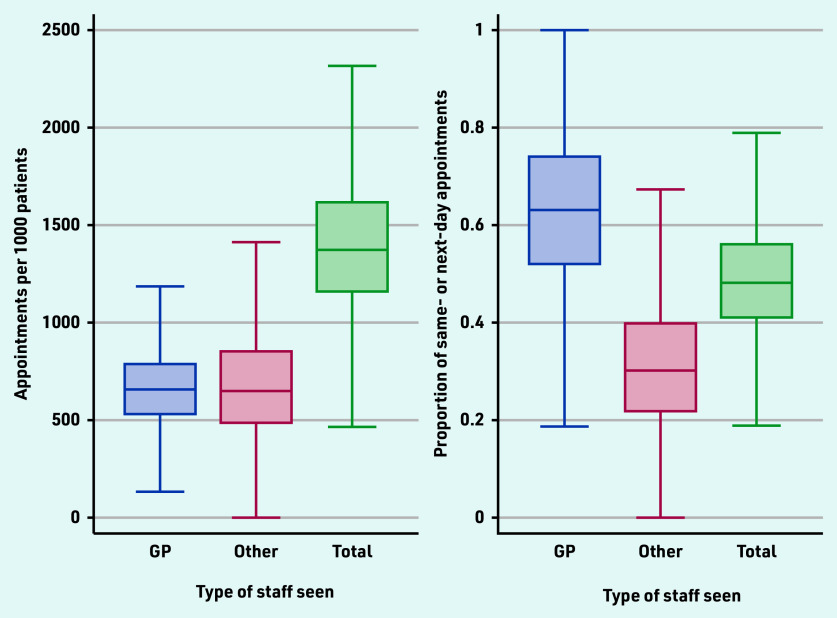
*Practice-level variation in appointment rates and proportions of appointments seen the same day or next day after booking.*

On average, practices delivered 1414 appointments per 1000 registered patients in the 3 months, August–October 2022 (Figure 1, Supplementary Table S1). There was substantial variation between practices in the volume of appointments per 1000 registered patients (10th percentile: 983; 25^th^ percentile: 1156; 75^th^ percentile: 1621 90^th^ percentile: 1895). There was also considerable variation in staffing, with an average of 0.58 FTE GPs per 1000 registered population (10th percentile: 0.27; 90^th^ percentile: 0.93), 0.26 FTE nurses (10^th^ percentile: 0.08; 90^th^ percentile: 0.46), 0.24 FTE other care professionals (10^th^ percentile: 0; 90^th^ percentile: 0.52), and 1.19 FTE administrative staff (10^th^ percentile: 0.73; 90^th^ percentile: 1.67). On average, 49% (41 918 125/84 945 202) of appointments were seen on the same or next day of booking. This was higher for GP appointments at 63% (25 261 756/39 801 536).

**Figure 2.**
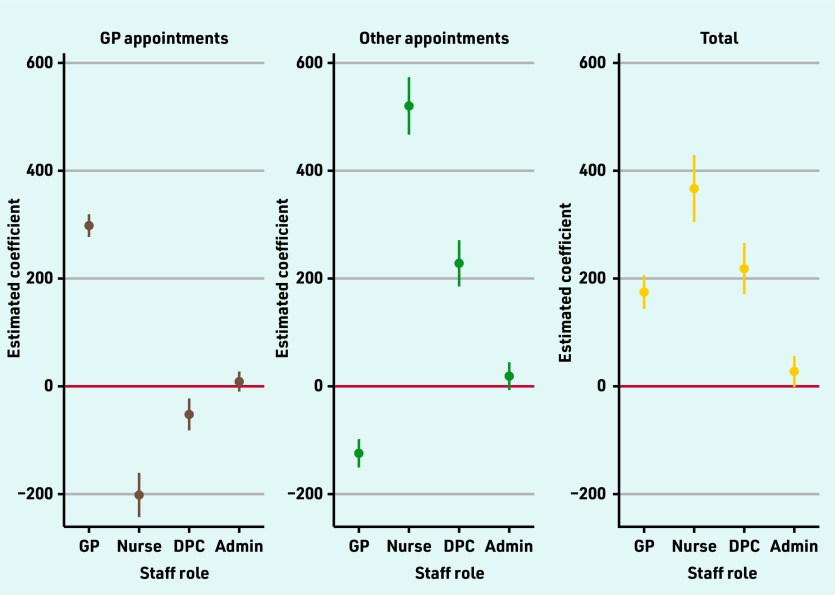
*Estimated effects of one additional full-time equivalent staff on numbers of appointments per 3 months.^a^* *^a^See Supplementary Table S2 for coefficients and 95% confidence intervals from multivariable regression models, including population and organisational characteristics. DPC = direct patient care.*

An additional FTE GP was associated with 175 (95% confidence interval [CI] = 144 to 206) more appointments over the 3 months (Figure 2). Additional FTEs of other clinical staff were associated with larger differences in 3-month appointment volumes of 367 (95% CI = 305 to 429) per additional FTE nurse, and 218 (95% CI = 171 to 266) per additional FTE other care professional (Supplementary Table S2).

**Figure 3.**
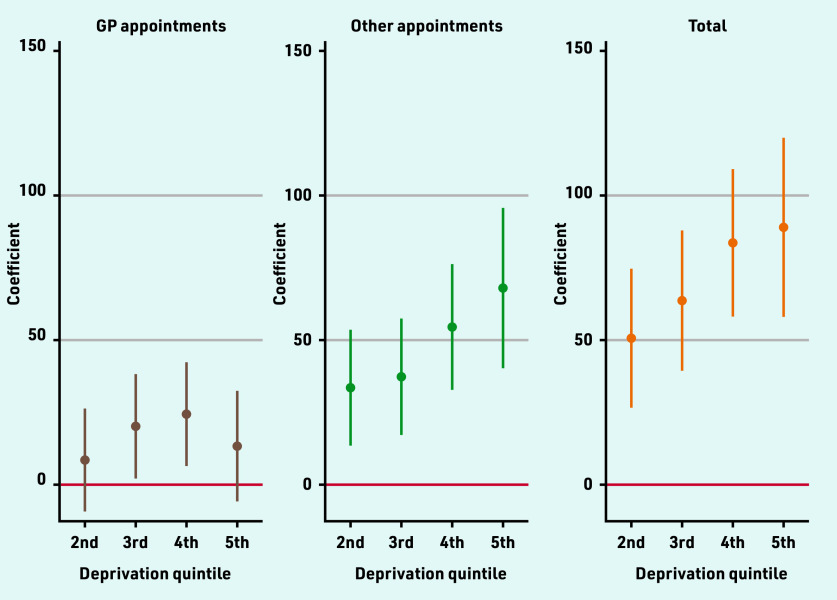
*Estimated differences in appointment rates between deprivation quintiles.^a^* *^a^ Estimated differences from the 1^st^ (least deprived) quintile. See Supplementary Table S2 for coefficients and 95% confidence intervals from multivariable regression models including population and organisational characteristics.*

Practices serving more deprived populations provided significantly more appointments, with practices in the middle quintile providing 64 (95% CI = 39 to 88; *P*<0.001) and practices in the most deprived quintile (quintile 5) providing 89 (95% CI = 58 to 120; *P*<0.001) more appointments per 1000 patients over 3 months compared with practices in the lowest deprivation quintile (Figure 3). Practices located in rural areas provided 47 (95% CI = 19 to 76) more appointments per 1000 registered population over 3 months than other practices. Practices on Personal Medical Services (PMS) contracts also had higher (28; 95% CI = 10 to 46) appointment rates, while dispensing practices had lower (−35; 95% CI = −65 to −4) appointment rates (Supplementary Table S2).

As expected, when appointments with specific staff types were examined, it was found that more GPs was associated with more appointments with GPs. One additional FTE GP was associated with 298 (95% CI = 277 to 319) more GP appointments over the 3 months. Higher FTE of other clinical staff were also associated with more appointments with non-GP staff. One FTE additional nurse was associated with 520 (95% CI = 467 to 574) and one additional FTE other care professional was associated with 228 (95% CI = 185 to 271) additional appointments per 3 months with these staff groups (Supplementary Table S2, Figure 2).

Evidence of substitution between different types of clinical staff was found since more GPs was associated with lower (−124; 95% CI = −150 to −98) appointment rates with other professionals and more other clinical staff were associated with lower appointment rates with GPs (nurses: −202 [95% CI = −243 to −161]; other care professionals: −52 [95% CI = −82 to −23]) over the 3 months (Figure 2, Supplementary Table S2).

While there was considerable variation in the proportion of appointments that occurred on the same or next day after booking (Figure 1), the associations between levels of staffing and speed of availability were less clear (Supplementary Table S3). There were significant associations only for appointments with other clinical staff; more FTE GPs was associated with a lower (−0.070 [95% CI = −0.084 to −0.056; *P*<0.001]) proportion of appointments occurring on the same or next day, and more FTE other staff (nurses: 0.090 [95% CI = 0.061 to 0.118; *P*<0.001], other care professionals: 0.049 [95% CI = 0.029 to 0.070; *P*<0.001]) associated with a higher proportion of appointments seen on the same or next day after booking.

Higher levels of deprivation were associated with higher proportions of appointments seen on the same or next day, especially for GP appointments where there was a 4.2 (95% CI = 2.6 to 5.9) percentage point (pp) difference between the highest and lowest deprivation quintiles. PMS practices had lower (−1.7pp [95% CI = −2.8 to −0.7]) proportions of GP appointments seen quickly, and rural practices had lower (−1.9pp [95% CI = −3.2 to −0.7]) proportions of appointments with other staff seen quickly (Supplementary Table S3).

In the supplementary analysis, the results were not affected materially by inclusion of the 120 practices with very low or very high appointment rates (Supplementary Table S4). The results were also unaffected by exclusion of practices with more than 10% missing data on staff type or very low or high rates of staff-specific appointments (Supplementary Table S5 and S6). The analysis of proportions of appointments seen on the same or next day after booking was also unaffected by exclusion of practices with poorer-quality data.

## DISCUSSION

### Summary

Improving primary care access is a key policy priority in England, and the government has pledged to increase appointment availability through expansion of workforce skill mix. However, the organisational factors associated with appointment volumes are unknown, especially the degree to which other care practitioners contribute to volumes and act as substitutes for GPs.

Newly available data were analysed to investigate the population, workforce, and organisational factors associated with appointment volumes. The study found that patients registered with practices with more staff per 1000 population have more appointments. It also found that the variations between practices follow expected patterns in terms of appointments by staff type, with more GPs associated with more GP appointments and more other staff associated with more appointments with other staff. The study found substitution between staff types in appointment volumes, because numbers of appointments delivered by other staff groups was lower in practices with higher numbers of FTE GPs per 1000 patients (and vice versa).

In terms of additional appointment volumes per FTE, the study found that nurses were associated with the highest number of additional appointments, followed by other direct patient care professionals. Grossing up to annual figures would suggest one additional FTE GP would be associated with 1193 additional appointments with GPs or a net of 700 total additional appointments per year, after accounting for staff substitution in the provision of appointments. One additional FTE nurse or other care practitioner would be associated with a net of 1468 and 874 additional appointments per year, respectively.

### Strengths and limitations

To the authors’ knowledge, this is the first analysis of the new national practice-level appointments dataset for England. Some previous studies have used national data from surveys but relied on self-reported measures of access, such as estimated time since last appointment. Others have analysed data extracted from practice records but have been restricted to using selected subsets of voluntarily participating practices and often faced tight restrictions on the locational and organisational information they could access. In the present study, some key factors for all practices in England were able to be considered, taking advantage of the substantial variability in workforce and organisational form that exists for primary care providers. Further analyses were undertaken to check sensitivity to outliers and potentially poor-quality data.

The main limitations of the analysis result from data availability. Appointments data are published as practice counts so patterns could not be looked at for individual patients or different types of patients. At this stage, it is only possible to estimate cross-sectional rather than longitudinal associations.

The appointments in general practice data that were examined is a new release, classified by NHS Digital as experimental statistics owing to variations in the quality of some data items.^10^ For example, owing to an issue with how information on healthcare professional type was extracted from practice systems, NHS Digital is only able to classify appointments as occurring with either GPs or ‘other practice staff’. While more granular workforce data were able to be used to examine how four different staff groups contributed to overall appointment volumes, the authors were only able to break down who these appointments were actually delivered by using this crude dichotomy of GP versus all other practice staff. NHS Digital identified data quality issues with the recording of appointment mode and duration owing to differences in local recording practices and systems, and so that information was not used in the analyses. While there were initially concerns about the coverage of the data during its earlier development, the August–October 2022 release that was analysed covers 98.7% of practices in England and 99.8% of all registered patients. It should be noted that the appointments captured in the data represent the lower bound of true practice activity levels, because certain activities, including appointments funded by enhanced access schemes, are not included.

### Comparison with existing literature

The levels of activity and associations with population characteristics are broadly consistent with previous studies that have used Clinical Practice Research Datalink (CPRD), The Health Improvement Network (THIN), and QRESEARCH.^15^^–^18 However, these were only able to analyse subsets of practices (<10% of total practices) meaning the results are likely to be less statistically robust. The present study found an average appointment rate of 1.414 appointments per patient during the 3 months examined, which would equate to 5.66 appointments per year. This is broadly in line with consultation rates estimated using CPRD, which have increased over time, from 4.67 in 2007–2008 to 5.16 in 2013–2014.^15^ QRESEARCH data showed decreases in the proportion of patients seen by a GP over time, from 77% in 1995 to 62% in 2006.^18^ This has further decreased to 47% in the present study’s dataset. The THIN dataset showed women living in more deprived areas had higher consultation rates.^17^ The present study’s regression results showed a similar gradient.

The finding that practices serving more deprived populations provided significantly more appointments per registered patient adds to previous findings of an inverse relationship between deprivation and consultation length.^19^ The present study was unable to consider appointment duration or complexity because these are not consistently measured between different practice system suppliers. However, NHS Digital is working to improve data on these areas,^10^ and this would enable future research to examine these competing elements of appointment provision together for the first time.

The study found higher levels of all clinical staff groups were associated with higher levels of appointments, a direct measure of patient access. This differs slightly from previous research that has shown that, while higher levels of GPs are associated positively with most outcomes, numbers of nurses and other care professionals is negatively associated with patient-reported access and satisfaction.^6^^,^^7^

### Implications for research and practice

The analysis has provided an interesting snapshot of appointments activity and future work should track changes over time. Future research should also seek to explain these differences, especially why deprived populations have more appointments with other care professionals but not with GPs. The consequences for patient and staff health and wellbeing should be evaluated.

An important implication from the study is that the published data may be more useful and of better quality than some people feared. The practice variations have shown many expected patterns and have highlighted well-known inequalities in workforce between practices^20^^–^22 lead to inequalities in the amount of contact patients receive.

Relieving pressure on GPs is often presented as the main policy goal for skill-mix expansions.^23^ The results on substitution between staff types suggest this is possible, but prior research suggests GPs’ job satisfaction and ability to delegate work were not associated with higher levels of other staff. In addition, while higher numbers of staff other than GPs are associated with higher appointment volumes and therefore improved access, previous research has suggested they are negatively associated with patient-reported access and satisfaction.^6^^,^^7^ While access is an important dimension of healthcare quality, the safety and effectiveness of care delivered must also be considered.^24^ Increasing appointment volumes has come at the cost of reduced continuity.^4^ Together these findings mean that caution is needed when pursuing increased access through skill-mix expansion, as this may come at the price of lower quality.

## Data

All datasets used in this study are publicly available. The links are included in the reference list.
